# Color stability, surface, and physicochemical properties of three-dimensional printed denture base resin reinforced with different nanofillers

**DOI:** 10.1038/s41598-024-51486-w

**Published:** 2024-01-22

**Authors:** Min-Chae Kim, Da-Jung Byeon, Eo-Jin Jeong, Hye-Bin Go, Song-Yi Yang

**Affiliations:** 1 Department of Dental Hygiene, Konyang University, 158 Gwanjeodong-ro, Seo-gu, Daejeon, 35365 Republic of Korea; 2 Department and Research Institute of Dental Biomaterials and Bioengineering, Yonsei University College of Dentistry, 50-1 Yonsei-ro, Seodaemun-gu, Seoul, 03722 Republic of Korea

**Keywords:** Health care, Medical research, Materials science

## Abstract

Various materials have been introduced for the three-dimensional (3D) printing of dentures. In this study, the color stability and surface and physicochemical properties of 3D-printed denture base resins with four types of nanofiller particles were evaluated. Al_2_O_3_, ZnO, CeZr, and SiO_2_ nanofillers were added to a 3D printable denture base-resin matrix and subjected to digital light processing. The specimens were immersed in Coke, coffee, black tea, or distilled water for 6 days. For the assessment of color differences, 6 samples were analyzed using a spectrophotometer. In a separate investigation, surface properties of 10 samples were examined, while a different set of 6 samples was used to analyze water sorption and solubility. All experimental groups exhibited higher color stability in Coke than the control group. However, the groups containing ZnO and CeZr had lower color stability in coffee and black tea than the control group. Moreover, they had agglomerated nanofillers and lower gloss than the control group. Compared with that of the control group, the contact angle of the CeZr group and microhardness of the ZnO group were not significantly different. Water sorption was higher in the Al_2_O_3_ group, whereas the solubility of the experimental and control groups was not statistically significant. The results demonstrated the significant effect of ZnO and CeZr nanofillers on the color stability of the dentures when exposed to discoloring beverages. These results will facilitate the development of fillers that enhance the resistance of 3D printed denture base resins to discoloration in the oral environment.

## Introduction

In the past decades, complete and partial dentures have been used to restore the function and aesthetics in edentulous patients. Polymethylmethacrylate (PMMA) is the preferred material for fabricating removable dentures owing to its low cost, ease of manipulation, stability in the oral environment, and satisfactory appearance^1^. Despite these advantages, PMMA has several drawbacks, including hypersensitivity, susceptibility to discoloration, abrasion, and potential porosity. Moreover, the presence of voids on both the surface and subsurface of the PMMA-based dentures can degrade their mechanical, aesthetic, and hygienic properties^2,3^.

With the development of digital dentistry, computer-aided design/manufacturing systems have been designed to address the limitations associated with traditional dental prosthetics^4^. These systems enable the manufacture of three-dimensional (3D) printed dentures with various advantages, such as reduced fabrication time, increased precision, cost-effectiveness, fewer patient visits, and improved patient comfort^5,6^. Digital light processing (DLP) is a liquid-based 3D printing technology that has been widely utilized in dental applications. It involves the use of ultraviolet or visible light to activate the photopolymerization process, resulting in the gradual layer-by-layer hardening of the photopolymer solution^7^. In dentistry, 3D printers have been applied for DLP to manufacture a wide range of dental products, including orthodontic models, surgical guides, crowns, bridges, splints, and denture bases^8,9^. Nonetheless, as 3D printed dentures have only been recently developed in dentistry, thorough assessment of their mechanical, physicochemical, and aesthetic characteristics is required before they can be considered as feasible alternatives to traditional PMMA dentures.

Denture wearers with poor oral hygiene often experience denture-associated stomatitis because of the accumulation of dental plaque over prolonged periods^10^ and discoloration of denture resins due to dietary choices^11^. In particular, beverages, such as tea, coffee, and wine, stain denture acrylic resins^12,13^. As such, denture wearers need to follow proper denture cleaning procedures to prevent biofilm accumulation on denture surfaces^14^. The most commonly performed denture cleaning process is the use of a toothbrush and denture cleaner, which is simple, economical, and effective in removing biofilms^15^. However, this can significantly affect the wear and surface roughness of the denture, which can promote biofilm formation and staining, resulting in surface detail loss and decreased gloss^16,17^.

Color stability is a clinical requirement for dental materials, whereby a significant change in color indicates aging or damage^18–21^. The discoloration of dental prostheses can be prevented by improving the color stability of a material by incorporating materials that enhance its resistance to discoloration^22–24^. In a previous study, nanosized zirconium dioxide (ZrO_2_), titanium dioxide (TiO_2_), and silicon dioxide (SiO_2_) particles were added to PMMA and their color stabilities were evaluated by immersion in various discoloring beverages^24^. Nano-ZrO_2_ was shown to increase the color stability of PMMA, suggesting a method for preventing the discoloration of denture base resins.

Despite the importance of the physicochemical and aesthetic properties of materials, studies on preventing the discoloration of 3D printed denture resin have been insufficient. In addition, the 3D printed denture base resins reinforced with nanofillers depends on a fundamental understanding of polymerization, specifically degree of conversion (DC), along with consideration of surface properties and overall material interactions. Therefore, this study aims to evaluate the effects of different nanofillers on the color stability, dimensional accuracy, DC, surface microstructure, gloss, contact angle, microhardness, water sorption, and solubility of 3D printed denture base resins. The null hypothesis is that various types of nanofillers incorporated into 3D printing denture base resin will not affect the color stability, dimensional accuracy, surface, and physicochemical properties.

## Materials and methods

### Materials

Uncured 3D printing denture resin (NextDent Denture 3D+ , 3D Systems, Soesterberg, Netherlands) was used as the 3D printing denture base resin matrix. The 3D printing resin matrix comprised 75 wt.% ethoxylated bisphenol A dimethacrylate, 10–20 wt.% 7,7,9 (or 7,9,9)-trimethyl-4,13-dioxo3,14-dioxa-5,12-diazahexadecane-1,16-diyl bismethacrylate, 5–10 wt.% 2 hydroxyethyl methacrylate, 5–10 wt.% SiO_2_, 1–5 wt.% diphenyl(2,4,6-trimethylbenzoyl)phosphine oxide, and < 0.1 wt.% TiO_2_. Four different types of nanofillers, namely aluminum oxide (Al_2_O_3_), zinc oxide (ZnO), cerium–zirconium oxide (CeO_4_Zr), and SiO_2_ powders, were individually added to the uncured 3D printing resin matrix at various concentrations (Table 1). These nanoparticles of size ≤ 50 nm were purchased from Sigma Aldrich (St. Louis, MO, USA). They were mixed with the uncured 3D printing resin matrix using a speed mixer (DAC 150.1 FVZ, Hauschild, Germany) at 3500 rpm for 2 min. Each mixture was stored in a dark environment at 23 ± 2 °C to prevent polymerization until the 3D printing process.

**Table 1:** Components and proportions of the control and experimental groups used in this study (wt.%).

Group (wt.%)	Composition				
	3D printing resin	Al_2_O_3_	ZnO	CeO_4_Zr	SiO_2_
Control	100.0	–	–	–	–
2.5% Al	97.5	2.5	–	–	–
5.0% Al	95.0	5.0	–	–	–
2.5% Zn	97.5	–	2.5	–	–
5.0% Zn	95.0	–	5.0	–	–
2.5% Ce	97.5	–	–	2.5	–
5.0% Ce	95.0	–	–	5.0	–
2.5% Si	97.5	–	–	–	2.5
5.0% Si	95.0	–	–	–	5.0

### Specimen preparation

Specimens of different shapes were designed for each experiment prior to the 3D printing process. Disk-shaped specimens of thickness 1.0 mm and diameter 10.0 mm were prepared for the color-stability measurement. The specimen for the dimensional accuracy measurement was a rod of dimensions 2.0 mm (height) × 2.0 mm (width) × 25.0 mm (length). The specimens for the scanning electron microscopy (SEM)–energy dispersive X-ray spectroscopy (EDS), surface gloss, DC, water sorption, and solubility measurements were disks of diameter 15.0 mm and thickness 1.0 mm. The specimens used for the contact angle and Vickers hardness measurements had dimensions of 64.0 mm × 10.0 mm × 3.3 mm. Each designed specimens were 3D printed using a DLP type 3D printer unit (NextDent 5100, 3D systems, Vertex Dental BV, Soesterberg, Netherland) with a layer thickness of 50 µm and orientation of 0°. The 3D printed specimens were removed from the platform and cleaned with isopropyl alcohol. The cleaned specimens were post-cured for 30 min using a 3D print box (NextDent LC-3D print box, 3D systems, Vertex Dental BV, Soesterberg, Netherlands) and polished with a water-cooled rotating polishing machine (Ecomet, Buehler Ltd., Lake Bluff, IL, USA) using 1,500-and 2,000-grit silicon carbide papers (Deerfos, Incheon, Korea).

### Color stability

The discoloration solution was obtained by mixing 2.7 g coffee (KANU Mild, Dongseo Foods, Seoul, Korea) and 100 mL boiling water for 10 min; this was followed by filtering the solution using a 190-micron paint strainer (Norton, Scotland, UK). Black tea was prepared in three tea bags (Lipton, Glasgow, England) seeped in 100 mL hot water for 10 min and filtered similarly. Coke (Coca-Cola Company, Atlanta, CA, USA) was used as received. Distilled water was used as the immersion solution for comparison. Twenty-four specimens per group were divided into four subgroups and individually immersed in coke, coffee, black tea, and distilled water at 37 ± 1 °C for 6 d. The discoloration solutions were replaced daily with fresh solutions.

The CIELAB coordinates of each specimen were determined using a spectrophotometer (CM-3500d; Konica Minolta, Tokyo, Japan), according to the SCE method. The baseline colors of the specimens were measured before immersion in the discoloration solutions. After immersion in the discoloration solution, the specimens in each solution were removed, washed with water, and dried in air for 15 s. All specimens were measured on a white background, and three randomly selected sites within each specimen were averaged and set as representative values to obtain the lightness (L^*^), and color opponents of green–red (a^*^) and blue–yellow (b^*^). The color change parameters, ∆L^*^, ∆a^*^, and ∆b^*^ were calculated using the equations:

1 $$\Delta L^{*} = L^{*} \left( {{\text{Experimental}}\,{\text{value}}} \right){-}L^{*} \left( {{\text{Baseline}}\,{\text{value}}} \right)$$

2 $$\Delta a^{*} = a^{*} \left( {{\text{Experimental}}\,{\text{value}}} \right){-}a^{*} \left( {{\text{Baseline}}\,{\text{value}}} \right)$$

3 $$\Delta b^{*} = b^{*} \left( {{\text{Experimental}}\,{\text{value}}} \right){-}b^{*} \left( {{\text{Baseline}}\,{\text{value}}} \right)$$

Consequently, the total color difference value (ΔE^*^) was calculated as follows:

4 $$\Delta E^{*} = \surd (\Delta L^{ * } )^{{2}} + (\Delta a^{ * } )^{{2}} + \, (\Delta b^{ * } )^{{2}}$$

### Dimensional accuracy

Ten specimens per group were printed. The supports of the 3D-printed specimens were not removed. Subsequently, the specimens were washed and cured. All specimens were measured at their middle point and both endpoints using digital Vernier calipers with an accuracy of 0.01 (Mitutoyo Corp, Kanogawa, Japan). The differences between the designed and printed sizes were calculated to evaluate the dimensional accuracy of the width (x axis), length (y axis), and height (z axis) of the specimens.

### DC

The DC was measured using Fourier-transform infrared spectroscopy (FTIR; Nicolet iS10, Thermo Scientific, MA, USA) at a resolution of 4 cm^−1^ and scanning range of 4000–500 cm^−1^. A drop of the uncured experimental resin mixed with the nanofiller was used to measure the DC of the uncured experimental resin composites. Subsequently, the DC values of the cured specimens were measured. After obtaining the baseline spectra, both the polymerized and unpolymerized, aromatic C=C bonds at 1608 cm^−1^, and aliphatic C=C bonds at 1638 cm^−1^ were used. The DC was determined using the equation:

5 $$DC\left(\%\right)=\left(1-\frac{{\left(1638 {{\text{cm}}}^{-1}/1608 {{\text{cm}}}^{-1}\right)}_{polymerized}}{{\left(1638 {{\text{cm}}}^{-1}/1608 {{\text{cm}}}^{-1}\right)}_{unpolymerized}}\right) \times 100$$

### SEM–EDS

Field-emission scanning electron microscopy (FE-SEM; Carl Zeiss, Oberkochen, Germany) was used to analyze the distribution of the nanofillers in the specimens. The specimens were observed at 500 × and 1,000 × by FE-SEM for the surface and component evaluation, respectively. Subsequently, EDS was performed to further analyze the distribution of the nanofillers in the specimen. Two sites were randomly analyzed for each specimen.

### Gloss

Prior to the test, the gloss meter was calibrated by comparing it with a calibration tile. The surface gloss of the specimen in gloss unit (GU) was measured at three different points, namely the middle and both sides, using a gloss meter (Novo-Curve, Rhopoint Instruments, East Sussex, UK). The measurements from three randomly selected sites within each specimen were averaged and set as representative values.

### Contact angle

The surface wettability evaluation of the specimens was measured using a droplet analysis device (Smartdrop, Femtofab, Gyeonggi-do, Korea), which is a contact angle measurement device. The sessile drop method was used to measure a drop of 5 µL distilled water on the surface of the specimen. The static contact angle was randomly measured twice for each sample surface, averaged, and set as a representative value.

### Vickers hardness

The Vickers hardness number was measured using a microhardness tester (MMT-X; Matuzawa, Akita, Japan) under a load of 300 g for 30 s. The measurements from three randomly selected sites within each specimen were averaged and set as representative values.

### Water sorption and solubility

Water sorption and solubility tests were performed according to ISO 20,795–1:2013 standards. All specimens were placed in a desiccator maintained at 37 ± 1 °C for 23 ± 1 h. The specimens were then stored in a desiccator maintained at 23 ± 2 °C for 60 ± 10 min and weighed with an analytical balance (XS105; Mettler-toledo AG, Greifensee, Switzerland) with an accuracy of ± 0.01 mg. The initial mass (*m*_1_) was measured repeatedly until the weight loss of < 0.2 mg. The diameter was measured thrice and the thickness was measured five times to calculate the volume (*V*) per specimen. The diameters and thicknesses of the specimens were measured using a digital caliper with an accuracy of 0.01 mm (Mitutoyo Model CD-15CPX; Mitutoyo Corporation, Kawasaki, Japan). Each sample was stored in distilled water at 37 ± 1 °C, blotted until free from visible moisture, dried in air for 15 s, and weighed (*m*_2_) within 60 s after being removed from the water. Finally, the specimens were kept in a desiccator maintained at 23 ± 2 °C and weighed daily until a constant dry mass (*m*_3_) was obtained. The water sorption and solubility were calculated using the following equations:

6 $$W_{{{\text{sp}}}} = (m_{{2}} - m_{{3}} )/V$$

7 $$W_{{{\text{sl}}}} = (m_{{1}} {-}m_{{3}} )/V$$

where *W*_*sp*_ is the water sorption of the test material (µg/mm^3^), and *W*_*sl*_ is the water solubility of the test material (µg/mm^3^).

### Statistical analysis

Statistical analyses were conducted using IBM SPSS Statistics (version 25.0; IBM Corp., Armonk, NY, USA). A one-way ANOVA was employed to compare color stability, dimensional accuracy, DC, gloss, contact angle, Vickers hardness, water sorption, and solubility of 3D printed denture base resins reinforced with different nanofillers. Significant findings from ANOVA were further analyzed using Tukey’s HSD test for pairwise comparisons. Statistical significance was set at a 95% confidence interval, with a *p*-value less than 0.05 considered significant.

## Results

### Color stability

**Figure 1:**
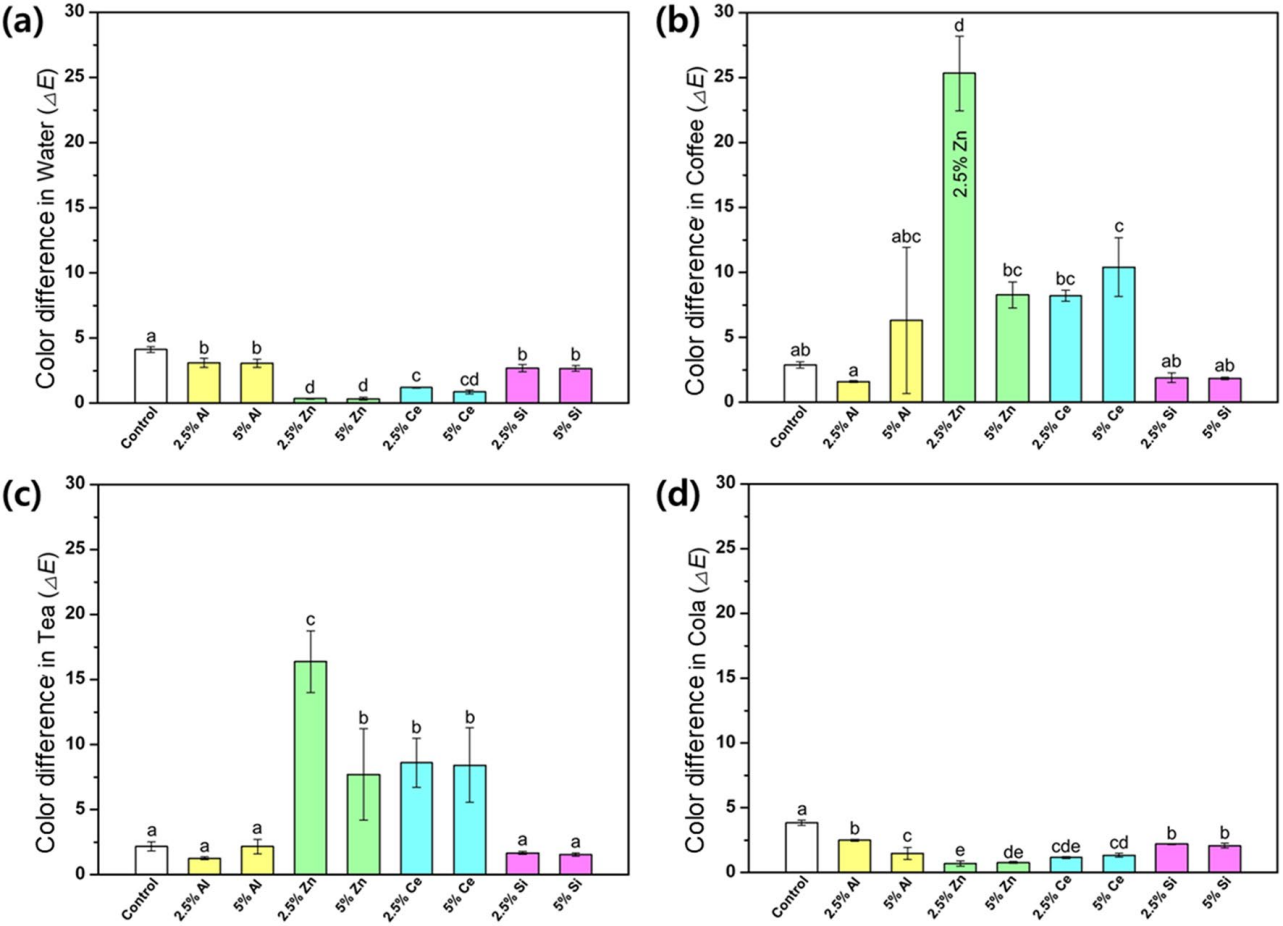
Color changes of the 3D printing denture base resin containing different types of nanofillers after immersion in (**a**) water, (**b**) coffee, (**c**) black tea, and (**d**) Coke (n = 6). The lowercase letters above the bars indicate the significant differences between groups (*p* < 0.05).

The color change on the specimen surface after immersion in various discoloration solutions is shown in Fig. 1. After soaking in coffee, the groups containing Al and Si showed no significant difference in color change compared with the control group (*p* > 0.05). However, the groups with 2.5% Zn and 5.0% Ce showed significantly higher color changes than the control group (*p* < 0.05), with 2.5% Zn showing the highest color change (*p* < 0.05). After immersion in the black tea solution, the 2.5% Al, 5.0% Al, 2.5% Si, and 5.0% Si groups showed statistically nonsignificant color changes compared with the control group (*p* > 0.05). Meanwhile, the experimental groups containing Zn and Ce showed a higher color change compared with the control group (*p* < 0.05) with the highest color change for the group containing 2.5% Zn (*p* < 0.05). After immersion in the Coke solution, all the experimental groups with different types of nanofillers showed significantly lower color changes compared with the control group (*p* < 0.05). Comparing the color stability after immersion in coffee and black tea, the groups containing Al and Si has higher color stability than the other experimental groups (*p* < 0.05). However, those with 2.5% Zn and 5.0% Ce had significantly lowered color stability after immersion in coffee and black tea than the other experimental groups (*p* < 0.05).

### Dimensional accuracy

The differences between the reference and measured data for the printed sample are listed in Table 2. The closer the calculated result is to zero, the better is the dimensional accuracy, indicating that there is no difference between the design and printed values. Meanwhile, a positive value indicates that the printed result is smaller than the set value, whereas a negative value indicates that the printed result is larger than the set value.

**Table 2:** Differences between the reference and measured data in the printed sample (mean ± SD).

Groups	X-axis (mm)	Y-axis (mm)	Z-axis (mm)
Control	0.03 ± 0.01^a^	− 0.01 ± 0.00^b^	− 0.02 ± 0.00^a^
2.5% Al	0.11 ± 0.01^a^	− 0.05 ± 0.00^ab^	0.01 ± 0.03^a^
5.0% Al	− 0.01 ± 0.01^a^	− 0.02 ± 0.00^ab^	− 0.04 ± 0.01^a^
2.5% Zn	0.23 ± 0.00^a^	− 0.06 ± 0.01^ab^	0.21 ± 0.00^bc^
5.0% Zn	0.22 ± 0.00^a^	− 0.01 ± 0.01^b^	0.27 ± 0.02^c^
2.5% Ce	0.14 ± 0.01^a^	− 0.06 ± 0.00^ab^	0.12 ± 0.01^b^
5.0% Ce	0.19 ± 0.00^a^	− 0.02 ± 0.00^b^	0.26 ± 0.00^c^
2.5% Si	0.04 ± 0.01^a^	− 0.07 ± 0.01^a^	0.01 ± 0.03^a^
5.0% Si	− 0.03 ± 0.00^a^	− 0.03 ± 0.00^ab^	− 0.08 ± 0.01^a^

The same lowercase letters in the same column indicate no significant differences between the groups (*p* > 0.05).

The difference between the set and printed values for the X-axis was not significant between the test and control groups (*p* > 0.05). Meanwhile, the difference between the set and printed values on the Y-axis was significant between the 2.5% Si and control groups (*p* < 0.05). Compared to the control group, there was a significant difference in the values of the 2.5% Zn, 5.0% Zn, 2.5% Ce, and 5.0% Ce groups (*p* < 0.05).

### DC

**Figure 2:**
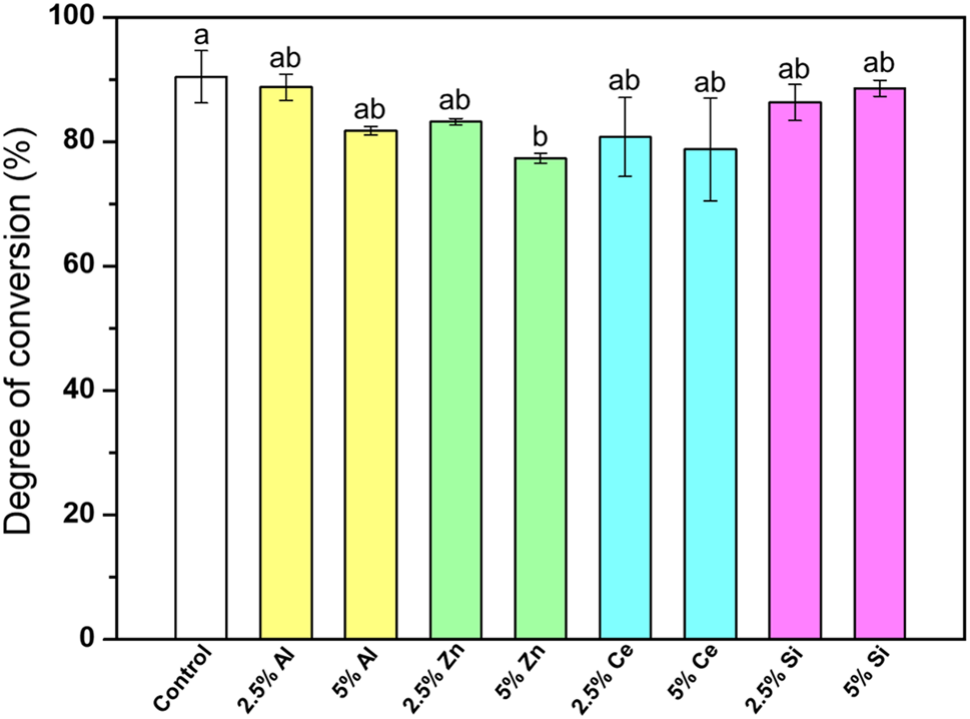
DC of the 3D printing denture base resin containing different types of nanofillers. The lowercase letters above the bars indicate significant differences between groups (*p* < 0.05).

The results of the DC analysis are shown in Fig. 2. All groups, except for the 5.0% Ce and 5.0% Zn groups showed a DC of 80% or higher. Compared to the control group, 5.0% Zn showed the lowest DC value, denoting a significant difference (*p* < 0.05).

### SEM–EDS

**Figure 3:**
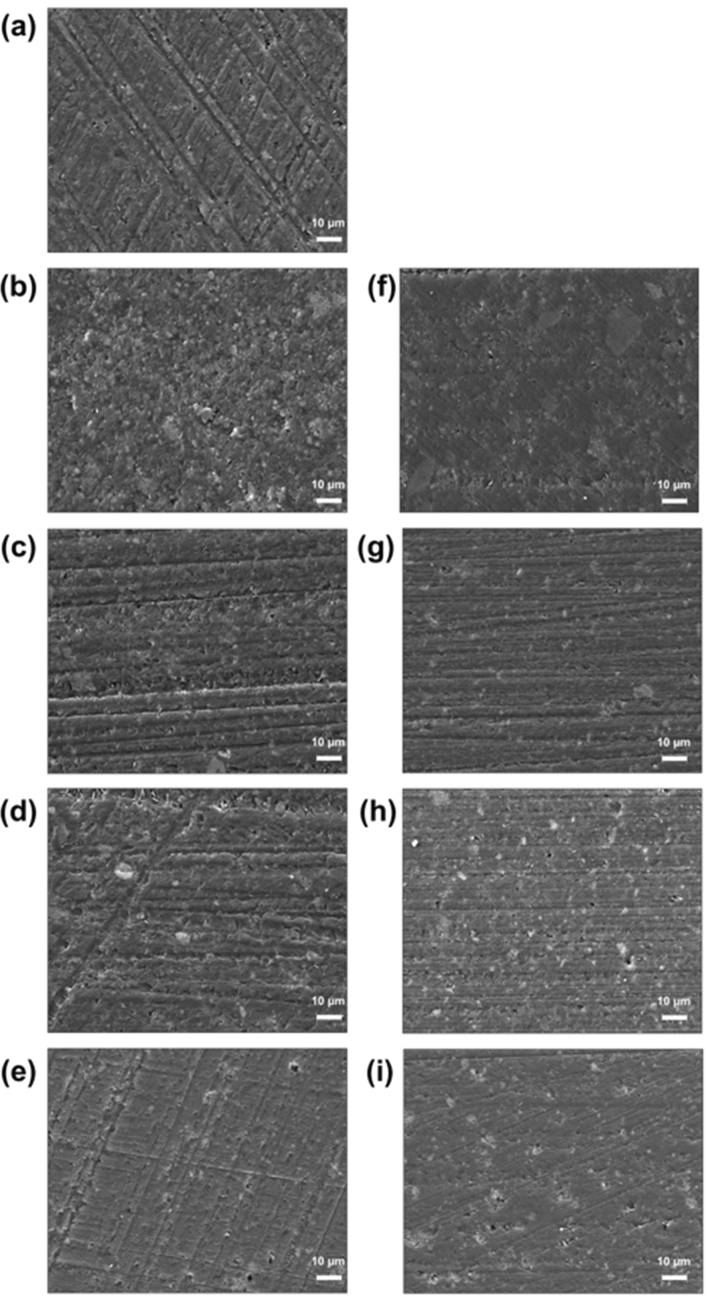
Representative SEM images of the 3D-printed denture resin surface containing different types of nanofillers at the magnification of 1000× : (**a**) control, (**b**) 2.5% Al, (**c**) 2.5% Zn, (**d**) 2.5% Ce, (**e**) 2.5% Si, (**f**) 5.0% Al, (**g**) 5.0% Zn, (**h**) 5.0% Ce, and (**i**) 5.0% Si.

Figure 3 shows the micromorphology of the printed specimen surface observed at a magnification of 1000× . Polishing traces were observed in all specimens, and the presence of the nanofillers was investigated. In the experimental groups containing 2.5% nanofillers, partially embedded nanofillers with traces of polishing were observed. The experimental groups containing 5% nanofillers exhibited more pronounced embedding of the nanofillers than the 2.5% group. Moreover, the control group exhibited more pronounced polishing traces.

**Figure 4:**
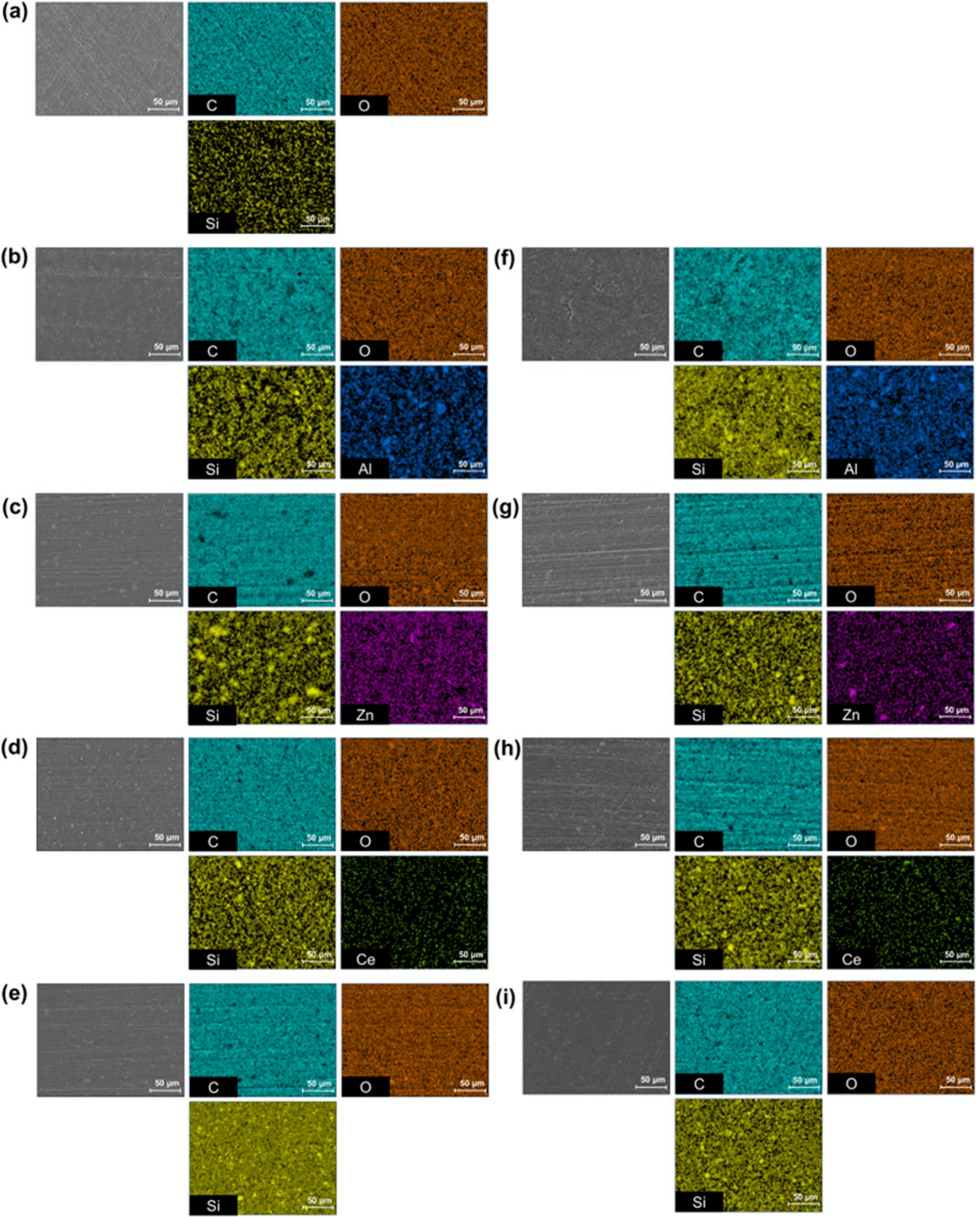
SEM–EDS results of the 3D-printed denture resin surface containing different types of nanofiller observed at the magnification of 500× : (**a**) control, (**b**) 2.5% Al, (**c**) 2.5% Zn, (**d**) 2.5% Ce, (**e**) 2.5 Si, (**f**) 5.0% Al, (**g**) 5.0% Zn, (**h**) 5.0% Ce, and (**i**) 5.0% Si.

The distribution of the different types of nanofillers was investigated by SEM–EDS, as shown in Fig. 4. Large amounts of organic materials, namely C and O, and small amounts of Si were detected in all specimens. As the nanoparticle content was increased, the number of spots on the surface increased. The nanofillers were added to the resin with a mostly even dispersion. However, in the 2.5% Al, 5.0% Al, and 2.5% Zn specimens, the nanofiller particles were slightly condensed.

### Gloss

**Figure 5:**
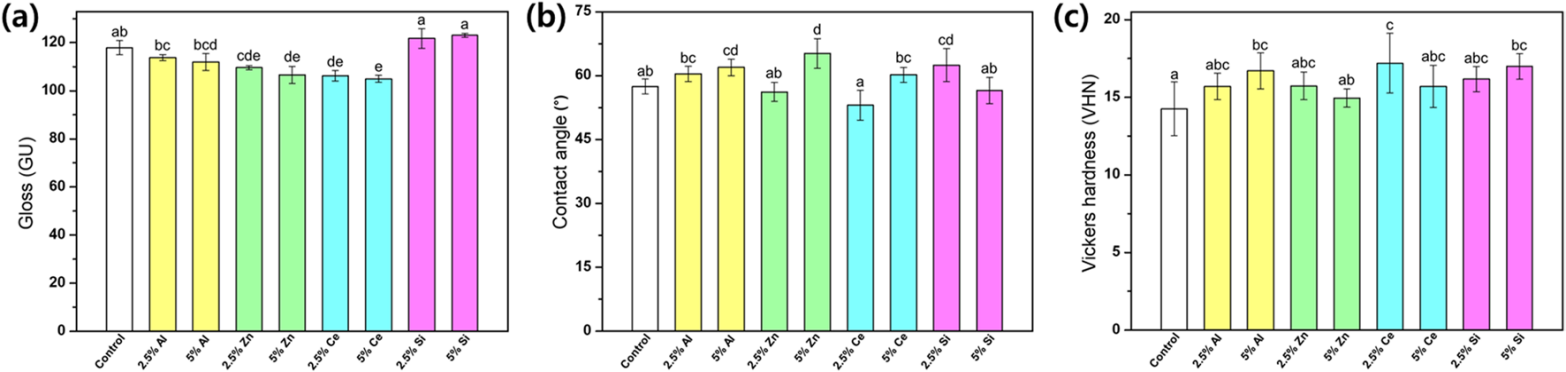
Mean and standard deviation for the (**a**) gloss, (**b**) contact angle, and (**c**) Vickers microhardness of the 3D-printed denture resins containing different types of nanofillers (n = 10). The lowercase letters above the bars indicate significant differences in each group (*p* < 0.05).

The results of the gloss measurement are shown in Fig. 5a. The groups containing Si and Al nanoparticles showed no significant differences compared to the control group (*p* > 0.05). However, the 5.0% Zn, 2.5% Zn, 5.0% Ce, and 2.5% Ce groups showed a significantly lower gloss than the control group (*p* < 0.05). In particular, the 5.0% Ce sample exhibited the lowest value.

### Contact angle

The contact angle measurements are shown in Fig. 5b. The 5.0% Zn group showed the highest contact angle, followed by the 2.5% Si and 5.0% Al groups with a statistically significant difference compared with the control group (*p* < 0.05).

### Surface microhardness

The Vickers microhardness results are shown in Fig. 5c. The 2.5% Ce group (17.20 ± 1.93) showed the highest hardness value, and the 2.5% Ce, 5.0% Si, and 5.0% Al were significantly different from the control group (*p* < 0.05).

### Water sorption and solubility

The water sorption and solubility results are presented in Table 3. Water sorption was significantly higher in the 5.0% Al group compared than in the control group (*p* < 0.05); however, the other values were not statistically significant (*p* > 0.05). The water solubilities of the 5.0% Zn, 5.0% Ce, and 2.5% Ce groups were higher than that of the control group but the differences between the groups were not statistically significant (*p* > 0.05).

**Table 3:** Water sorption and solubility of the control and experimental groups (mean ± SD).

Groups	Water sorption (μg/mm^3^)	Water solubility (μg/mm^3^)
Control	24.48 ± 0.61^b^	0^a^
2.5% Al	25.61 ± 2.15^ab^	0^a^
5.0% Al	28.22 ± 2.98^a^	0^a^
2.5% Zn	24.55 ± 1.57^b^	0^a^
5.0% Zn	26.82 ± 1.68^ab^	0.87 ± 0.79^a^
2.5% Ce	24.19 ± 1.51^b^	0.45 ± 1.09^a^
5.0% Ce	26.39 ± 1.31^ab^	0.50 ± 0.85^a^
2.5% Si	24.04 ± 0.95^b^	0^a^
5.0% Si	25.40 ± 0.75^ab^	0^a^

The same lowercase letter in the same column indicates a statistically insignificant difference between groups (*p* > 0.05).

## Discussion

The discoloration of denture resins can be attributed to multiple factors, including surface abrasion, inadequate oral hygiene, beverage-induced staining, and water absorption, which can lead to dissatisfaction of the wearer and eventual replacement^11^. In this study, we fabricated specimens using 3D printing denture base resins with various nanofillers and immersed them in beverages that are known to cause discoloration. The discoloration solutions were chosen based on the beverage used to evaluate the color stability of dental materials with an immersion time of 6 d. This period was intended to simulate a 24-h storage period as one month to determine the discoloration caused by six months of beverage consumption^24^. The evaluation of color stability after immersion in coffee and black tea solutions revealed notable differences, particularly in the 2.5% Al, 2.5% Si, and 5.0% Si groups, which were not significantly different from the control (*p* > 0.05), whereas the Zn and Ce group showed a significantly higher color difference (*p* < 0.05). Moreover, we observed a significant difference in the color change between the specimens in the coffee and black tea solutions, which is consistent with previous findings of greater discoloration in coffee solutions^25,26^. Conversely, after immersion in the Coke solution, all experimental groups exhibited lower color differences than the control group (*p* < 0.05). Although the discoloration effect of Coke on the specimens in this study was relatively modest compared to that of coffee or black tea, the specimens with nanofillers exhibited superior color stability compared to the control group. The relatively milder discoloration effect of Coke compared with other beverages may be attributed to a phenomenon in which accumulated layers that detach from the sample surface are subsequently returned to the solution^24^. The analysis of the color difference showed the lowest values for 2.5% Al, which was closely followed by 5.0% Si. In clinical dentistry, the amount of clinically acceptable color change (*∆E*) has been established to be 3.3; changes beyond this threshold is considered unacceptable^27,28^. In our study, all experimental groups, including the 2.5% Al, 2.5% Si, and 5.0% Si groups, exhibited *∆E* values below 3.3 when exposed to coffee and black tea solutions. After immersion in the Coke solution, all experimental groups, except the control group, showed Δ*E* values below 3.3. Therefore, the color changes observed in the 2.5% Al, 2.5% Si, and 5.0% Si groups were within clinically acceptable ranges for all solutions. From a clinical perspective, the incorporation of a small amount of Si or 2.5% Al into the 3D printing denture base resin was proven to be an effective strategy for reducing discoloration, potentially contributing to prolonged denture usage and increased patient satisfaction.

Dimensional accuracy is a major requirement for 3D printing resins and a critical factor in the clinical success of custom-made dental devices^29^. In this study, we employed a Vernier caliper to precisely gauge the variance between the design and output values. The results of the dimensional accuracy test showed no significant differences between the experimental and control groups on the X-axis (*p* > 0.05). However, significant differences were noted on the Y-axis for the 2.5% Si group compared with the control group (*p* < 0.05). The factors that affect the shrinkage of 3D-printed resins, include filler content, monomer structure, filler/matrix interactions, additives, and polymerization factors (polymerization rate, catalyst and inhibitor concentration, and curing method)^30^. Considering these factors, measurements with Vernier calipers along with the comparison of the test data with reference models using color difference maps and 3D measurement software are necessary to obtain accurate and reliable dimensional measurements for evaluating the dimensional stability of newly developed materials. The Z-axis results showed that the experimental groups containing Zn and Ce showed a significant difference compared to the control group (*p* < 0.05). These results suggest that the presence of Zn and Ce in the nanofillers induces a high surface energy, promoting particle agglomeration, resulting in significant differences observed along the Z axis^31,32^.

It is important to ensure the even dispersion of nanofillers within the 3D printing resin. If the nanofillers are not evenly dispersed, the particles can agglomerate, thereby degrading the mechanical properties of the composite material^33^. The SEM–EDS observations showed the agglomeration of nanofillers when the Zn and Ce concentrations were increased to 5.0%. As such, the use of nanofillers treated with silane coupling agents on the surface is recommended to ensure uniform and efficient dispersion and minimize the aggregation of these nanofillers^34^.

When new materials are incorporated into denture materials, the noticeable loss of gloss has a significant impact on their clinical usability^35^. The Si-containing groups exhibited relatively high gloss values, whereas the 2.5% Zn, 5.0% Zn, and 5.0% Ce groups exhibited lower gloss values. This is attributed to the increased surface roughness owing to the presence of nanofiller agglomerates^36^. These results are consistent with the more pronounced agglomeration of the Zn and Ce nanofiller particles compared to the other experimental groups based on the SEM–EDS images.

The DC is an important factor that determines the polymerization rate of a resin material. Low polymerization rates of resin materials lead to poor mechanical and physical properties, and high cytotoxicity^37,38^. Various methods can be used to measure the DC, including Raman spectrophotometry and FT-IR spectroscopy. Among these, FT-IR spectroscopy has a fairly high spectral signal-to-noise ratio, high wavenumber accuracy, sensitivity, and high scanning speed with high resolution, providing useful information, such as peak position, intensity, width, and shape^39^. Using this method, we demonstrated that the composition or content of the nanofiller did not significantly affect the polymerization rate of the 3D printing resin, except for the 5.0% Zn group. However, previous studies have reported that different factors, such as the shape, size, and content of the filler can also affect the DC results^40^.

The contact angle measurements provide information on the wettability of a surface, which is related to its properties^41^. Previous studies have noted that surface roughness tends to increase when the solid surface is hydrophilic, suggesting that an increase in surface wettability leads to a color change^42^. In this study, the measured contact angles increased with increasing nanofiller content in all experimental groups, except for the Si group. Additionally, the 5.0% Zn, 2.5% Si, and 5.0% Al groups displayed significantly elevated contact angles compared to the control group (*p* < 0.05).

The high water sorption of denture base resins can affect the development of internal stresses and crack formation in the intraoral environment, affecting the mechanical properties and color stability of dentures^43^. Along with water absorption, water solubility changes the shape, volume, and quality of dental materials in the oral cavity, making it an important indicator for durability assessment^44^. In this study, there was no significant difference in the water absorption and solubility among the groups. Moreover, 5.0% Al showed the highest water absorption at 28.22 µg/mm^3^. This finding is similar to previous results showing that the addition of Al_2_O_3_ nanofillers to PMMA slightly increases the water absorption and solubility than those of the control^45^. Nevertheless, the water absorption and solubility values of all the experimental groups used in this study were below the ISO 20,795–1:2013 (Dentistry—Base polymers—Part 1: Denture base polymers) standards of 32 and 1.6 µg/mm^3^, respectively, suggesting their suitability as denture base materials.

The surface hardness measurements showed that the 2.5% Ce and 5.0% Si groups had significantly higher hardness values than the control group (*p* < 0.05). These results demonstrate the influence of nanoparticles on the surface hardness of 3D printing resins. Higher surface hardness enhances wear resistance and reduces possible damage to the material by brushing or eating hard foods^46^.

However, this study has several limitations. First, our investigation only explored the addition of nanoparticles to one type of 3D printing denture base resin matrix, which may limit the comprehensiveness of our findings. Additionally, the 3D printing resin substrate utilized in this study contained a small amount of Si, which may have influenced our observations owing to variations in the nanofiller composition. To overcome these limitations, additional investigations are required to examine 3D printing resins from various manufacturers or to utilize a resin matrix that is specifically designed for 3D printing without inorganic filler components. Despite these limitations, this study demonstrated the notable effects of different nanofillers in enhancing the color stability, surface properties, and physical and chemical properties of 3D printing resins. Further, these findings may be useful for future applications in denture manufacturing by 3D printing.

## Conclusions

In this study, different types of nanofillers were mixed into a 3D printing denture base resin, which was subsequently printed using a DLP printer. The color stability and surface and physicochemical properties of the resulting materials were evaluated. The conclusions obtained in this work are as follows:

1. All experimental groups exhibited enhanced color stability in the Coke solution compared with the control group. Conversely, in coffee and black tea solutions, the experimental groups containing Zn and Ce demonstrated significantly reduced color stability compared to the control group.
2. In the dimensional accuracy tests, the experimental and control groups showed no significant differences in terms of the X-axis dimensions. However, for the Y-axis dimensions, the 2.5% Si group showed significantly different values from the control group. Moreover, significant differences were noted for the Z-axis dimensions of the experimental groups containing Zn and Ce compared with the control group.
3. Except for the 5.0% Zn group, none of the experimental groups exhibited a significant difference in DC compared with the control group.
4. The SEM–EDS images revealed the partial embedding of nanofillers on the surface, along with polishing traces. Notably, the nanofillers exhibit partial agglomeration, particularly in the experimental groups containing Zn and Ce.
5. Surface gloss was significantly lower in the groups containing Zn and Ce than in the control group, whereas the contact angle was significantly higher in the 5.0% Al, 5.0% Zn, and 2.5% Si groups than in the control. In addition, the surface microhardness was significantly enhanced in the 5.0% Al, 2.5% Ce, and 5.0% Si groups compared to the control group.
6. Water sorption was significantly higher in the 5.0% Al group than in the control, whereas solubility showed no significant difference between the experimental and control groups.

This study demonstrates the significant effect of 3D-printed denture resins containing Zn and Ce nanofillers on color stability when exposed to discoloration-causing beverages, such as coffee and black tea. Different types of nanofillers significantly affected the polymerization rate and surface and physicochemical properties of the material. These findings are expected to serve as a useful reference for the selection and formulation of nanofillers to improve the color stability of 3D printed denture base resins in the oral environment.

## Data Availability

The data will be made available by the corresponding author upon reasonable request.
